# Standard *versus* distal Roux-en-Y gastric bypass in patients with BMI 50–60 kg/m^2^: 5-year outcomes of a double-blind, randomized clinical trial

**DOI:** 10.1093/bjsopen/zrab105

**Published:** 2021-11-17

**Authors:** Odd Bjørn Kjeldaas Salte, Marius Svanevik, Hilde Risstad, Dag Hofsø, Ingvild Kristine Blom-Høgestøl, Line Kristin Johnson, Morten Wang Fagerland, Jon Kristinsson, Jøran Hjelmesæth, Tom Mala, Rune Sandbu

**Affiliations:** Department of Gastrointestinal and Paediatric Surgery, Oslo University Hospital, University of Oslo, Oslo, Norway; Department of Gastrointestinal Surgery, Vestfold Hospital Trust, Norway; Department of Endocrinology, Morbid Obesity and Preventive Medicine, Oslo University Hospital, Oslo, Norway; Morbid Obesity Centre, Vestfold Hospital Trust, Norway; Department of Endocrinology, Morbid Obesity and Preventive Medicine, Oslo University Hospital, Oslo, Norway; Morbid Obesity Centre, Vestfold Hospital Trust, Norway; Oslo Centre for Biostatistics and Epidemiology, Research Support Services, Oslo University Hospital, Oslo, Norway; Department of Endocrinology, Morbid Obesity and Preventive Medicine, Oslo University Hospital, Oslo, Norway; Morbid Obesity Centre, Department of Medicine, Vestfold Hospital Trust, Tønsberg, Norway; Department of Gastrointestinal and Paediatric Surgery, Oslo University Hospital, University of Oslo, Oslo, Norway; Department of Gastrointestinal Surgery, Vestfold Hospital Trust, Norway

## Abstract

**Background:**

The optimal surgical weight loss procedure for patients with a BMI of 50 kg/m^2^ or more is uncertain. This study compared distal Roux-en-Y gastric bypass (RYGB) with standard RYGB.

**Methods:**

In this double-blind RCT, patients aged 18–60 years with a BMI of 50–60 kg/m^2^ were allocated randomly to receive standard (150 cm alimentary, 50 cm biliopancreatic limb) or distal (150 cm common channel, 50 cm biliopancreatic limb) RYGB. The primary outcome (change in BMI at 2 years) has been reported previously. Secondary outcomes 5 years after surgery, such as weight loss, health-related quality of life, and nutritional outcomes are reported.

**Results:**

Between May 2011 and April 2013, 123 patients were randomized, 113 received an intervention, and 92 attended 5-year follow-up. Mean age was 40 (95 per cent c.i. 38 to 41) years and 73 patients (65 per cent) were women; 57 underwent standard RYGB and 56 distal RYGB. BMI was reduced by 15.1 (95 per cent c.i. 13.9 to 16.2) kg/m^2^ after standard and 15.7 (14.5 to 16.9) kg/m^2^ after distal RYGB; the between-group difference was −0.64 (−2.3 to 1.0) kg/m^2^ (*P* = 0.447). Total cholesterol, low-density lipoprotein cholesterol, and haemoglobin A1c levels declined more after distal than after standard RYGB. High-density lipoprotein cholesterol levels increased more after standard RYGB. Vitamin A and vitamin D levels were lower after distal RYGB. Changes in bone mineral density, resting metabolic rate, and total energy intake were comparable.

**Conclusion:**

Distal RYGB did not enable greater weight loss than standard RYGB. Differences in other outcomes favouring distal RYGB may not justify routine use of this procedure in patients with a BMI of 50–60 kg/m^2^. Registration number: NCT00821197 (http://www.clinicaltrials.gov).

Presented in part as abstract to the IFSO (International Federation for the Surgery of Obesity and Metabolic disorders) conference, Madrid, Spain, August 2019.

## Teaser

The optimal surgical weight loss procedure for patients with a BMI of 50 kg/m^2^ or more is uncertain. This study was designed to determine whether distal Roux-en-Y gastric bypass (RYGB) enables greater weight loss than standard RYGB.

## Introduction 

Bariatric surgery may ensure significant weight loss and improved health in patients with severe obesity^1^. Roux-en-Y gastric bypass (RYGB) has shown good long-term outcomes with regard to weight loss, co-morbidities, and health-related quality of life (HRQoL)^2^.

BMI exceeds 50 kg/m^2^ in a large subset of individuals with severe obesity^3^. More than half of these patients may have a BMI of over 40 kg/m^2^ 5 years after standard RYGB^4^. Greater weight loss is achieved after biliopancreatic diversion or duodenal switch, but increased malabsorption may cause nutritional deficiencies and diarrhoea^4^^,^^5^.

A RYGB is typically constructed with an alimentary limb (Roux limb) for gastrojejunal bypass of about 100–150 cm. Increasing malabsorption by lengthening the alimentary limb has been suggested as a means of increasing weight loss, but no firm conclusions have yet been made^6–9^.

An RCT^10^ with two profoundly different lengths of alimentary limb, and with a fixed biliopancreatic limb length, was conducted; a standard RYGB (alimentary limb 150 cm) was compared with a distal RYGB (very long alimentary limb with a common channel of 150 cm). If the principle of elongation of the alimentary limb in RYGB promotes greater weight loss, this study would reveal the differences. However, no differences in weight loss were observed after 2 years.

This follow-up study investigated whether the distal RYGB would increase weight loss at 5 years. Adverse events, nutritional outcomes, HRQoL, cardiometabolic risk factors and gastrointestinal side-effects were also evaluated. Finally, mechanisms of weight loss were explored by evaluation of energy intake and energy expenditure.

## Methods

### Trial design and participants

A double-blind, parallel-group, RCT was performed. Between May 2011 and April 2013, all patients referred for bariatric surgery aged 20–60 years with BMI of 50–60 kg/m^2^ were assessed for study inclusion at two public tertiary-care institutions in Norway (Oslo University Hospital and Vestfold Hospital Trust). Exclusion criteria comprised previous bariatric or major abdominal surgery, kidney stones, chronic liver disease, and conditions associated with poor compliance. Five-year follow-up was completed by September 2018. Details of the study design have been reported previously^11^. Permuted-block randomization was undertaken. Eligible patients were assigned randomly to standard or distal RYGB in a 1 : 1 allocation ratio. Patients, follow-up study personnel at the outpatient clinics, and the statistician were unaware of the treatment allocation.

The study was approved by the Regional Ethics Committees for Medical and Health Research and registered in ClinicalTrials.gov (NCT00821197). All patients provided written and informed consent.

### Interventions and follow-up

Both procedures were performed using an antegastric, antecolic Roux-en-Y configuration with a gastric pouch of about 25 ml and a biliopancreatic limb of 50 cm. In standard RYGB, the alimentary limb length was 150 cm from the gastrojejunostomy to the jejunojejunostomy^11^. A common channel length of 100 cm or more is assumed necessary to reduce the risk of nutritional deficiencies related to malabsorption^12^. In distal RYGB, the jejunoileostomy was therefore established 150 cm from the ileocaecal junction. Vitamin and mineral prescriptions were identical for both groups, and adjusted during follow-up according to defined algorithms^11^. Follow-up consultations were scheduled at 6 weeks, 6 months, and 1, 2, and 5 years after surgery. Bodyweight and body composition (fat mass, fat-free mass) were measured to the nearest 0.1 kg (Tanita^®^-BC 418 MA; Tanita Corporation, West Drayton, UK) with light clothing and no shoes.

### Definitions of co-morbidities and adverse events

Medical conditions, medical visits, hospital admissions, operations, medications, and supplements were registered at the consultations according to the patient’s own reports using predefined standard case record forms, and relevant data were retrieved from available medical files. Definitions of medical conditions can be found in *Table S1*. All adverse events requiring intervention up to 5 years were recorded.

### Dual-energy X-ray absorptiometry

Dual-energy X-ray absorptiometry (DXA) scans were performed of the lumbar spine (L1–L4), left hip, and left proximal femur at both centres. At Vestfold Hospital Trust, all patients were examined with a Hologic^®^ Delphi W instrument (Hologic^®^, Bedford, MA, USA); at Oslo University Hospital, a GE Lunar Prodigy (General Electric Company^©^, Chicago, IL, USA)was used until 26 August 2016, when it was replaced by a GE Lunar iDXA. Cross-calibration of the two DXA scanners has been published previously^13^. Areal bone mineral density (aBMD), t-scores, and z-scores were calculated with enCore version 17 software (GE Medical Systems^©^, Madison, WI, USA) based on a large database of reference populations from the NHANES I–III and Lunar studies provided by the manufacturer. The database contains data from healthy adults and allows correction of measured values based on age, sex, and ethnicity^14^.

### Blood samples

Blood samples were drawn after an overnight fast. Vitamins, lipids, bone markers (carboxyl terminal telopeptide of type 1 collagen (CTX), procollagen type 1 N-terminal propeptide, and bone-specific alkaline phosphatase) were analysed by the hormone laboratory at Oslo University Hospital.

### Patient reported outcome measures

Generic HRQoL was evaluated using the Short Form 36 Health Survey (SF-36^®^) version 1 and 2 (4-week recall) survey at baseline, and 1, 2 and 5 years after surgery^15^. SF-36^®^ was scored using Health Outcomes scoring software version 5.1 (Optum^®^, Eden Prairie, MN, USA). Obesity-related quality of life was assessed using Obesity and Weight-Loss Quality of Life (OWLQOL) and Weight Related Symptom Measure (WRSM) questionnaires at 1-, 2-, and 5-year follow-up^16^^,^^17^.

At the 5-year follow-up, the Hospital Anxiety and Depression Scale (HADS)^18^^,^^19^, the generic Three-Factor Eating Questionnaire-R 21^20^^,^^21^, the Gastrointestinal Symptoms Rating Scale (GSRS)^22^, and a separate bowel function questionnaire reporting faecal incontinence and constipation were completed by the patients^22^^,^^23^. Dietary intake was evaluated by the Food Frequency Questionnaire self-report, estimating the percentage energy intake from protein, fat, carbohydrate, and alcohol^24^. The HADS questionnaire was translated to depression (HADS-D) and anxiety (HADS-A) domains. A cut-off point of 8 or more yields an adequate sensitivity and specificity for clinically relevant symptoms of depression or anxiety^19^.

### Resting metabolic rate

After 5 years, study participants at Vestfold Hospital Trust were offered examination of resting metabolic rate (Metalyzer^®^, Cortex 2; Biophysik, Leipzig, Germany). Patients met after overnight fasting at 08.00 hours and had been instructed to avoid physical exercise for 48 h. In a mixing chamber, oxygen, carbon dioxide, and ventilation were analysed every 10 s. The patients were lying relaxed at 45° in a dark quiet environment for 30 min. The exhaled air was analysed continuously through a facemask (V2; Hans Rudolph, Shawnee, KS, USA) and data were calculated from 15 min in the middle of the test.

### Statistical analysis

It was estimated that 88 patients would ensure a power of more than 80 per cent to detect a difference in BMI between the study groups of 3.0 kg/m^2^ 2 years after surgery (primary endpoint). To allow for potential lost to follow-up, 113 patients were included in total^11^.

Linear mixed models were fitted to all continuous variables with three or more repeated measurements. The models included fixed effects for treatment group, time, and treatment × time interaction, and a random intercept. Following model fit, mean values, changes from baseline, and between-group differences in changes from baseline were estimated with 95 per cent confidence intervals. Comparisons of non-repeated continuous variables were made using independent-samples *t* test and Mann–Whitney *U* test as appropriate. Adverse events and other categorical variables were analysed with chi^2^ tests and Fisher’s mid-*P* tests (sparse data)^25^. Statistical analyses were performed with Stata/SE^®^ version 16 (StataCorp, College Station, TX, USA), Matlab^®^ R2014a (Matrix Laboratory, The Mathworks Inc., Natick, MA, USA) and SPSS^®^ version 25 (IBM, Armonk, NY, USA). Missing data for patient-reported outcome measures were handled according to scale instructions. If no instructions were available, missing data were not imputed.

## Results

**Fig. 1:**
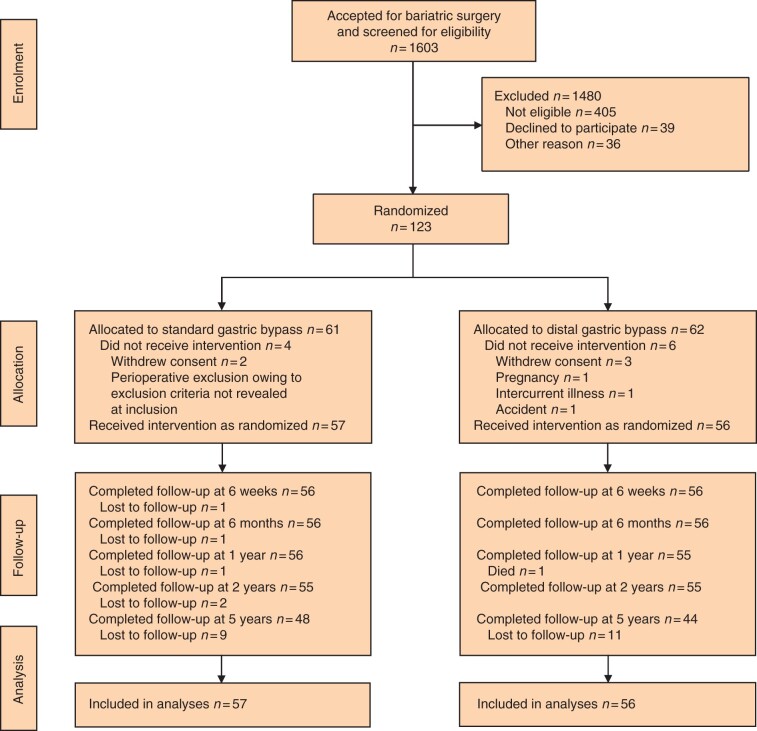
CONSORT diagram for trial of standard *versus* distal Roux-en-Y gastric bypass for patients with a BMI of 50–60 kg/m^2^

After 5 years, 92 of 113 patients (81 per cent) attended follow-up consultations; 48 (84 per cent) had undergone standard RYGB and 44 (79 per cent) distal RYGB (*Fig. 1*). Patient characteristics and demographics at baseline and follow-up are shown in *Table 1*. Four patients, two from each group, were deblinded during follow-up because of medical emergencies.

**Table 1:** Patient characteristics at baseline and at 5 years in the RCT of standard *versus* distal Roux-en-Y gastric bypass

	Baseline		5 years		***P*** §
	Standard RYGB	Distal RYGB	Standard RYGB	Distal RYGB	
	(*n* = 57)	(*n* = 56)	(*n* = 48)	(*n* = 44)	
**Demographics**					
Age (years)*	39.4 (9.3)	42.0 (8.2)	44.1 (9.6)	46.8 (7.7)	0.27
Women	36 (63)	37 (66)	31 (65)	29 (66)	0.89
Ethnicity, caucasian	57 (100)	55 (98)	48 (100)	43 (98)	0.29
General measures*					
Weight (kg)	160 (20)	157 (17)	114 (22)	110 (18)	0.54#
BMI (kg/m^2^)	53.3 (2.5)	53.6 (3.3)	38.2 (6)	37.9 (6)	0.45#
Systolic BP (mmHg)	131 (16)	138 (17)	127 (16)	126 (13)	0.01#
Diastolic BP (mmHg)	80 (11)	80 (12)	8 ( 9)	81 (8)	0.52#
**Medical conditions and medications**					
Diabetes mellitus type 2	14 (25)	19 (34)	1 (2)	2 (5)	0.42
Use of glucose-lowering medication	10 (18)	14 (25)	1 (2)	1 (2)	0.75
Insulin use	3 (5)	2 (4)	0 (0)	0 (0)	0.50
Diabetes resolution complete			6 (13)	10 (23)	0.20
Hypertension	33 (58)	34 (61)	11 (23)	12 (27)	0.63
Use of antihypertensive medication	22 (39)	26 (46)	7 (15)	6 (14)	0.90
Dyslipidaemia	46 (81)	50 (89)	19 (40)	14 (32)	0.44
Use of lipid-lowering medication	4 (7)	9 (16)	1 (2)	1 (2)	0.75
Metabolic syndrome	41 (72)	41 (73)	9 (19)	10 (23)	0.64
OSAS	21 (36)	19 (34)	1 (2)	3 (7)	0.23
CPAP-dependent OSAS	17 (30)	14 (25)	1 (2)	2 (4)	0.42
Joint pain	33 (58)	40 (71)	11 (23)	15 (34)	0.23
Depression	13 (23)	9 (16)	6 (13)	8 (18)	0.45
Urinary incontinence	10 (18)	13 (23)	3 (6)	2 (5)	0.83
Gastro-oesophageal reflux disease†	14 (25)	16 (29)	2 (4)	6 (14)	0.10
Hypothyroidism	3 (5)	11 (20)	1 (2)	6 (14)	0.03
Current smoker	8 (14)	14 (25)	4 (8)	1 (2)	0.28
Secondary hyperparathyroidism	12 (21)	12 (21)	18 (38)	26 (59)	0.04
Anaemia	5 (9)	5 (9)	8 (17)	7 (16)	0.92
Iron deficiency	11 (19)	11 (20)	12 (25)	18 (41)	0.10
Vitamin deficiencies (all)‡	35 (61)	36 (64)	17 (35)	21 (48)	0.23
Vitamin D deficiency	33 (58)	32 (57)	13 (27)	17 (39)	0.24

Values in parentheses are percentages unless indicated otherwise;

*values are mean (s.d.).

†Defined by use of proton pump inhibitors and/or described in medical record.

‡Deficiencies in vitamin A, vitamin B1 (thiamine), vitamin B2 (folate), B12, and/or 25-hydroxyvitamin D. RYGB, Roux-en-Y gastric bypass; OSAS, obstructive sleep apnoea; CPAP, continuous positive airway pressure.

§χ^2^ test (or Fisher mid-*P* test when number smaller than 5) for comparisons between groups at 5 years, except.

¶independent-samples *t* test and #mixed-model analysis of repeated measurements.

### Bodyweight and BMI

**Fig. 2:**
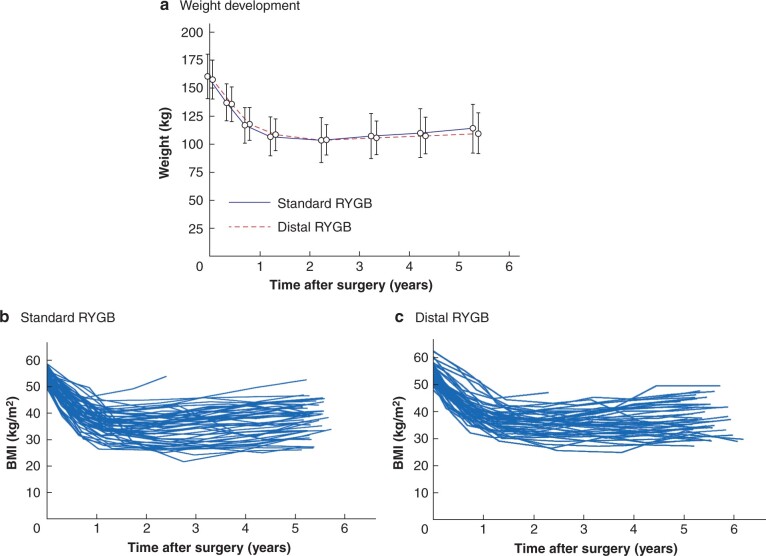
Weight development and BMI trajectories after surgery **a** Mean(s.d.) weight development in each group, and BMI trajectories for individual patients who underwent **b** standard or **c** distal Roux-en-Y gastric bypass (RYGB), over 5 years. At baseline, 57 patients were allocated to standard and 56 to distal RYGB. At study consultations 6 weeks, 6 months, and 1, 2, 3, 4 and 5 years after standard RYGB, 56, 56, 56, 55, 52, 49, and 48 patients attended follow-up. The corresponding figures for distal RYGB were 56, 56, 55, 55, 49, 43, and 44.

The mean reduction in BMI was 15.1 (95 per cent c.i. 13.9 to 16.2) kg/m^2^ after standard RYGB and 15.7 (14.5 to 16.9) kg/m^2^ after distal RYGB (*Table 2*). The mean between-group difference was −0.64 (−2.3 to 1.0) kg/m^2^ (*P* = 0.447) (*Table 2*). Mean percentage total weight loss was 28.9 (25.8 to 32.0) and 29.9 (26.8 to 32.9) per cent respectively. The mean between-group difference was −1.0 (−3.3 to 5.2) per cent (*P* = 0.66). Weight development and BMI trajectories for individual patients over 5 years are displayed in *Fig. 2*.

**Table 2:** Baseline, and 2- and 5-year outcomes after standard or distal Roux-en-Y gastric bypass

	Mean value			Mean change from baseline to 5 years	Mean between-group difference in changes from baseline to 5 years	*P* for between-group difference
	Baseline	2 years	5 years			
	(Standard *n* = 57)	(Standard *n* = 55*)	(Standard *n* = 48*)			
	(Distal *n* = 56)	(Distal *n* = 55*)	(Distal *n* = 44*)			
**Weight (kg)**						
Standard	160.2 (155.6, 164.8)	103.7 (99.1, 108.3)	114.4 (109.7, 119.2)	−45.7 (−49.3, −42.2)	−1.58 (−6.6, 3.5)	0.540
Distal	157.4 (152.7, 162.0)	103.6 (99.0, 108.3)	110.0 (105.2, 114.8)	−47.3 (−50.9, −43.7)		
**BMI (kg/m^2^)**						
Standard	53.3 (52.1, 54.6)	34.7 (33.5, 36.0)	38.2 (37.0, 39.5)	−15.1 (−16.2, −13.9)	−0.64 (−2.3, 1.0)	0.447
Distal	53.6 (52.4, 54.9)	35.5 (34.3, 36.8)	37.9 (36.6, 39.2)	−15.7 (−16.9, −14.5)		
**Systolic BP (mmHg)**						
Standard	131 (127, 135)	124 (120, 128)	127 (123, 131)	−4.2 (−8.1, −0.2)	−7.5 (−13.2, −1.8)	0.010
Distal	137 (133, 141)	128 (124, 132)	126 (121, 130)	−11.7 (−15.7, −7.6)		
**Diastolic BP (mmHg)**						
Standard	80 (77, 82)	77 (75, 80)	80 (78, 83)	0.6 (−2.1, 3.3)	−1.3 (−5.1, 2.6)	0.515
Distal	81 (79, 84)	80 (78, 83)	80 (78, 83)	−0.7 (−3.4, 2.1)		
**Glucose (mg/dl)**						
Standard	106.2 (99.0, 111.6)	88.2 (82.8, 95.4)	91.8 (86.4, 99.0)	−12.7 (−19.8, −7.2)	−10.1 (−18.7, −1.3)	0.025
Distal	113.4 (108.0, 118.8)	86.4 (81.0, 93.6)	90.0 (82.8, 97.2)	−23.4 (−30.6, −18.0)		
**HbA1c (%)**						
Standard	6.1 (5.8, 6.3)	5.3 (5.1, 5.5)	5.4 (5.2, 5.6)	−0.6 (−0.9, −0.4)	−0.3 (−0.6, −0.04)	0.03
Distal	6.2 (6.0, 6.4)	5.1 (4.9, 5.3)	5.2 (5.0, 5.4)	−1.0 (−1.2, −0.8)		
**HbA1c (mmol/mol)**						
Standard	41 (39, 43)	34 (32, 37)	36 (33, 38)	−5.2 (−7.2, −3.2)	−3.5 (−6.4, −0.6)	0.018*
Distal	42 (40, 44)	32 (30, 34)	33 (31, 36)	−8.7 (−10.8, −6.6)		
**Total cholesterol (mg/dl)**						
Standard	198.7 (191.0, 206.3)	166.3 (158.5, 174.0)	172.7 (164.6, 180.8)	−26.0 (−33.2, −18.8)	−38.2 (−48.6, −27.7)	< 0.001
Distal	203.7 (196.0, 211.5)	134.6 (126.8, 142.5)	139.6 (131.3, 147.9)	−64.1 (−71.6, −56.6)		
**HDL (mg/dl)**						
Standard	43.8 (40.8, 46.8)	59.9 (56.9, 63.0)	59.3 (56.1, 62.5)	15.5 (12.7, 18.3)	−5.9 (−10.0, −1.9)	0.004
Distal	44.7 (41.6, 47.7)	52.8 (49.7, 55.9)	54.2 (50.9, 57.5)	9.5 (6.6, 12.5)		
**LDL (mg/dl)**						
Standard	124.0 (117.4, 130.7)	88.5 (81.8, 95.3)	101.5 (94.5, 108.5)	−22.5 (−28.9, −16.2)	−32.2 (−41.3, −23.1)	< 0.001
Distal	128.4 (121.7, 135.1)	64.9 (58.1, 71.8)	73.6 (66.4, 80.9)	−54.7 (−61.3, −48.2)		
**Triglycerides (mg/dl)**						
Standard	156.5 (144.0, 169.1)	92.7 (79.9, 105.6)	99.2 (85.9, 112.6)	−57.3 (−70.1, −44.6)	−10.7 (−29.0, 7.6)	0.252
Distal	156.4 (143.7, 169.1)	86.6 (73.6, 99.6)	88.4 (74.6, 102.1)	−68.0 (−81.2, −54.9)		
**CRP (mg/dl)**						
Standard	1.3 (1.0, 1.5)	0.2 (0.0, 0.4)	0.2 (0.0, 0.4)	−1.1 (−1.4, −0.8)	−0.3 (−0.7, 0.2)	0.292
Distal	1.5 (1.3, 1.7)	0.2 (0.0, 0.5)	0.1 (0.0, 0.4)	−1.4 (−1.7, −1.0)		

Values in parentheses are 95 per cent confidence intervals. ^*^Attended follow-up. HbA1c, glycated haemoglobin; HDL, high-density lipoprotein; LDL, low-density lipoprotein; CRP, C-reactive protein. Linear mixed-model analysis for 113 patients included at baseline. Values for HbA1c were converted from percentages to mmoles per mole, then analysed with mixed models.

### Body composition

Mean fat mass at 5 years was 60.0 (95 per cent c.i. 54.9 to 65.1) kg for standard RYGB and 56.1 (51.0 to 61.2) kg for distal RYGB, with a mean difference between groups of 3.9 (−3.2 to 11.1) kg (*P* = 0.28). Mean fat free mass was 55.0 (49.1 to 60.8) and 52.8 (47.2 to 58.3) kg respectively, with a mean difference between groups of 2.2 (−5.8 to 10.2) kg (*P* = 0.59).

### Co-morbidity

The mean reduction in HbA1c and fasting glucose were greater after distal RYGB, as were mean levels of total and low-density lipoprotein (LDL) cholesterol. Mean high-density lipoprotein (HDL) cholesterol levels increased more after standard RYGB (*Table 2*). Co-morbidities at baseline and after 5 years are summarized in *Table 1*. There were no differences between groups in prevalence of type 2 diabetes, hypertension, dyslipidaemia or metabolic syndrome at 5 years (*Table 1*).

### Adverse events

Sixteen patients (28 per cent) had repeat abdominal surgery after standard RYGB and 10 (18 per cent) after distal RYGB (*P* = 0.20) (*Table 3*). Two patients with a distal RYGB underwent lengthening of the common channel owing to malabsorption, and in one patient the standard RYGB was reversed because of intractable hypoglycaemic episodes. One patient died from liver failure 12 months after distal RYGB.

**Table 3:** Adverse events requiring intervention up to 5 years after laparoscopic standard or distal Roux-en-Y gastric bypass

	Standard RYGB	Distal RYGB	***P*** ‡
	(*n* = 57)	(*n* = 56)	
**Patients with adverse events**	40 (70)	47 (84)	0.08
**Total no. of adverse events**	130	153	0.08
**Patients who had abdominal operations**	16 (28)	10 (18)	0.20
**No. of abdominal operations**	28	12	0.09
**Patients admitted to hospital for all reasons**	31 (54)	31 (55)	0.92
**No. of hospital admissions for all reasons**	52	56	0.78
**Patients with plastic surgical procedures**	5 (9)	4 (7)	0.75
**No. of plastic surgical procedures**	7	5	0.73
**Deaths**	0	1 (2)	0.50
Adverse events (0–30 days)*			
No adverse events	52 (91)	46 (82)	
Mild complications	4	2	
Pneumomediastinum	1	0	
Haematoma	1	1	
Haematochezia	0	1	
Superficial skin burn from warm liver retractor	1	0	
Hypertension	1	0	
Moderate complications	1	2	
Urinary tract infection	1	0	
Intra-abdominal abscess	0	1	
Melaena	0	1	
Severe complications	0	6	
Small bowel obstruction	0	2	
Intra-abdominal bleeding	0	1	
Leakage (enteroenteroanastomosis)	0	1	
Small bowel perforation	0	1†	
Ventral hernia recurrence	0	1	
Death	0	0	
**Adverse events (30 days to 5 years)**			
Gastrointestinal			
Internal herniation	8 (14)	1 (2)	0.02
Gastrojejunal ulcer	2 (4)	3 (5)	0.52
Small bowel obstruction	1 (2)	1 (2)	0.75
Incisional hernia	1 (2)	3 (5)	0.24
Acute liver failure	0	1 (2)	0.25
Cholecystitis, cholelithiasis, cholecystectomy	2 (4)	3 (5)	0.52
Appendicitis, appendicectomy	1 (2)	1 (2)	0.75
Acute abdominal pain	9 (16)	11 (20)	0.59
Chronic abdominal pain	9 (16)	7 (13)	0.62
Diarrhoea	4 (7)	12 (21)	0.03
Constipation	5 (9)	1 (2)	0.16
Nausea/vomiting	3 (5)	2 (4)	0.84
Gastro-oesophageal reflux disease	2 (4)	2 (4)	0.80
Oesophagitis	0	1 (2)	0.25
Other			
Hypoglycaemia	7 (12)	11 (20)	0.29
Urolithiasis	4 (7)	5 (9)	0.62
Infectious disease	17 (30)	13 (23)	0.43
Depression	4 (7)	3 (5)	0.86
Anxiety	3 (5)	4 (7)	0.58
Fatigue	1 (2)	2 (4)	0.43
Eating disorder	1 (2)	2 (4)	0.43
Alcoholism	1 (2)	1 (2)	0.75
Cancer	1 (2)	2 (4)	0.43
Arthrosis	3 (5)	3(5)	0.84
Other (not categorized)	21 (37)	20 (36)	0.90

Values in parentheses are percentages. *Severity of complications graded according to the contracted Accordion classification of 30-day complications after surgery. †The patient underwent a second laparotomy owing to bleeding after removal of an abdominal drain. RYGB, Roux-en-Y gastric bypass. ‡χ^2^ test or Fisher’s mid-*P* test (sparse data).

### Nutritional status

At the 5-year follow-up, vitamin A levels were reduced after distal RYGB only. 25-Hydroxyvitamin D levels increased after standard RYGB. Thiamine and folate levels increased after both procedures, with a greater increase after distal RYGB. Changes and developments in nutritional variables during the observation period are shown in *Table 4*. In total, 13 patients (27 per cent) who had standard RYGB and 17 (39 per cent) who underwent distal RYGB had 25-hydroxyvitamin D levels below the recommended value (less than 20 ng/mL) (*P* = 0.24). Twenty-six (54 per cent) and 23 (52 per cent) patients used oral nutritional supplementation 5 years after standard and distal RYGB respectively (*P* = 0.29).

**Table 4:** Nutritional measurements for patients before, and 2 and 5 years after standard or distal Roux-en-Y gastric bypass

	Mean value at baseline	Deficiency at baseline*	Mean value at 2 years	Mean value at 5 years	Deficiency at 5 years*	Mean change from baseline to 5 years	Mean between-group difference in changes from baseline to 5 years	*P* for between- group difference
	(Standard *n* = 57)		(Standard *n* = 55^†^)	Standard (*n *= 48^†^)				
	(Distal *n* = 56)		**(Distal *n* = 55** † **)**	**Distal (*n* = 44** † **)**				
**Vitamin A (µg/dl)**								
Standard	54.9 (51.6, 58.2)	0 (0)	50.7 (47.3, 54.0)	56.1 (52.6, 59.7)	0 (0)	1.2 (−2.4, 4.7)	−7.4 (−12.6, −2.2)	0.005
Distal	54.4 (49.8, 57.8)	0 (0)	50.7 (47.2, 54.1)	48.2 (44.4, 51.9)	0 (0)	−6.2 (−10.0, −2.4)		
**Vitamin D (ng/ml)**								
Standard	18.8 (16.8, 20.9)	35 (61)	23.0 (20.9, 25.2)	25.4 (23.2, 27.7)	13 (27)	6.6 (4.2, 9.1)	−4.4 (−8.0, −0.8)	0.016
Distal	18.5 (16.4, 20.5)	32 (57)	19.3 (17.2, 21.5)	20.7 (18.3, 23.0)	17 (39)	2.2 (−0.4, 4.8)		
**Vitamin B12 (pg/ml)**								
Standard	414 (305, 523)	0 (0)	802 (691, 913)	632 (513,752)	1 (2)	219 (67, 370)	−25 (−243, 192)	0.819
Distal	449 (339, 558)	0 (0)	700 (588, 812)	642 (517, 767)	1 (2)	193 (37, 349)		
**Vitamin B1 (thiamine) (nmol/l)**								
Standard	143 (135, 150)	0 (0)	153 (145, 160)	159 (151, 167)	0 (0)	16 (9, 23)	12 (2, 22)	0.021
Distal	147 (140, 155)	0 (0)	167 (159, 175)	175 (167, 183)	0 (0)	28 (21, 35)		
**Vitamin B9 (folate) (ng/ml)**								
Standard	4.9 (4.0, 5.9)	7 (12)	8.0 (7.0, 9.0)	7.4 (6.3, 8.4)	4 (8)	2.4 (1.4, 3.4)	1.6 (0.2, 3.1)	0.026
Distal	5.5 (4.5, 6.5)	9 (16)	10.0 (9.0, 11.0)	9.5 (8.5, 10.6)	4 (9)	4.0 (3.0, 5.1)		
**Haemoglobin (g/l)**								
Standard	140 (136, 144)	4 (7)	134 (131, 138)	137 (133, 141)	9 (19)	−3.1 (−7.0, 0.9)	−0.2 (−5.9, 5.5)	0.948
Distal	141 (137, 144)	3 (5)	137 (133, 141)	137 (133, 141)	6 (14)	−3.3 (−7.3, 0.8)		
**Ferritin (ng/ml)**								
Standard	151 (124, 180)	4 (7)	122 (94, 150)	78 (49, 108)	12 (25)	−73 (−103, −44)	17 (−26, 60)	0.439
Distal	142 (114, 170)	4 (7)	98 (69, 126)	85 (55, 116)	12 (27)	−56 (−87, −26)		
**Albumin (g/dl)**								
Standard	4.4 (4.3, 4.4)	0 (0)	4.3 (4.2, 4.4)	4.2 (4.1, 4.3)	2 (4)	−0.2 (−0.2, −0.1)	−0.1 (−0.2, 0.01)	0.069
Distal	4.3 (4.2, 4.4)	0 (0)	4.1 (4.1, 4.2)	4.0 (4.0, 4.1)	2 (4)	−0.3 (−0.3, −0.2)		

Values in parentheses are 95 per cent confidence intervals unless indicated otherwise:

*values in parentheses are percentages.

†Attended follow-up. Vitamin and mineral prescriptions were identical for both groups: oral daily one tablet of multivitamins, 1000 mg calcium carbonate, 800 units vitamin D3, and 65–200 mg iron. Intramuscular vitamin B12 was recommended every third month. Definitions of vitamin deficiencies: vitamin A, less than 10 µg/dl (less than 0.35 µmol/l) (refererence 20–80 µg/dl); vitamin B1 (thiamine), less than 70 nmol/l (reference 95–200 nmol/l); vitamin B9 (folate), less than 3 ng/ml (less than 7 nmol/l) (reference 340–1020 ng/ml); vitamin B12, less than 200 pg/ml (less than 150 pmol/l) (reference 200–1000 pg/ml); 25-hydroxyvitamin D, less than 20 ng/ml (50 nmol/l) (reference over 30 ng/ml), and/or increased substitution therapy. Linear mixed-model analysis for 113 patients included at baseline.

### Bone mineral density and serum bone markers

At 5-year follow-up, there were no between-group differences in aBMD (*Table S2*). Two patients were prescribed bisphosphonates after distal RYGB before follow-up. A third patient had a t-score below −2.5 in a lumbar vertebra and a compression fracture in Th12 after distal RYGB. Treatment with vitamin D was intensified and bisphosphonate treatment initiated. CTX-1 and parathyroid hormone levels, and the prevalence of secondary hyperparathyroidism increased more after distal RYGB (*Table 1* and *Table S3*).

### Patient-reported outcome measures

#### Quality of life, anxiety, and depression

The SF-36^®^ physical component summary scale scores improved from baseline to 5 years, with no differences between groups. The mental component summary scale scores remained unchanged (*Table S4* and *Fig. S1*). OWLQOL scores improved from baseline, with no difference between groups (*Table S4*). WRSM scores decreased comparably (*Table 4*), illustrating the decreased burden of obesity-related symptoms experienced by both groups. There were no between-group differences in anxiety or depression scores at follow-up (*Table S5*).

#### Eating behaviour

Eating behaviour scores did not differ between groups 5 years after surgery. Three patients had eating disorders during follow-up according to psychiatric evaluations (1 after standard RYGB, 2 after distal RYGB; *P* = 0.80) (*Table S5*).

#### Gastrointestinal symptoms

The GSRS questionnaire revealed more symptoms of diarrhoea 5 years after distal RYGB, with a mean score difference of −0.72 (95 per cent c.i. −1.32 to −0.13) between groups (*P* = 0.02) (*Table S6*). There were no differences in symptoms of indigestion, constipation, abdominal pain or reflux between the groups (*Table S6*).

#### Energy intake, distribution of energy-yielding nutrients, and resting metabolic rate

The mean total energy intake was 1952 (95 per cent c.i. 1648 to 2221) and 2341 (2002 to 2681) kcal/day after standard and distal RYGB respectively (*P* = 0.07) (*Table S7*). Percentage energy intakes from protein, fat, carbohydrate, and alcohol were comparable, although the absolute intake of protein was slightly higher after distal RYGB, with a mean between-group difference of 17 (3 to 32) g/day. Resting metabolic rate was assessed in 37 patients, with no between-group differences (*Table S8*).

## Discussion

It was hypothesized that elongation of the alimentary limb in RYGB would increase weight loss in patients with a BMI of 50–60 kg/m^2^. However, at the 5-year follow-up, no differences were found in BMI and percentage total weight loss between standard and distal RYGB. Differences in cardiovascular risk factors in favour of distal RYGB were observed. After distal RYGB, patients reported more frequent loose stools (diarrhoea), and two patients were reoperated with elongation of the common channel because of protein malabsorption.

With an average total small bowel length of about 6.5 m, the alimentary limb length of the distal RYGB would be about 3 m longer than that for the standard RYGB^8^. Most bariatric procedures have been designed based on an assumption that weight loss can be promoted through restriction of food intake, malabsorption or a combination of these mechanisms. However, physiological mechanisms that have an impact on hunger, meal satiety, food preferences, and energy expenditure may be more prominent contributors to weight loss^26^.

Biliopancreatic diversion with duodenal switch promotes weight loss by shortening the common channel and thereby challenges the physiological limits for macronutrient absorption. As the large difference in alimentary limb length in the study groups did not affect weight loss in the present study, the gastrojejunal bypass *per se*, and not the length of the alimentary limb, emerges as a plausible major contributing mechanism to weight loss after RYGB.

The authors^27^ have previously reported higher food and caloric intake after duodenal switch compared with standard RYGB. A previous report^28^ documented similar basal metabolic rates 4–7 years after either duodenal switch or standard RYGB in patients with a BMI of 50–60 kg/m^2^. Theoretically, patients could compensate for malabsorption after distal RYGB with increased food intake or a modified metabolic rate. To explore these mechanisms, approximate energy intake and resting metabolic rate were estimated after 5 years. It was observed that patients had slightly higher protein intake after distal RYGB; however, the total energy intake, although numerically higher after distal RYGB, was not statistically significant from that after standard RYGB. The resting metabolic rate was similar across the two groups, as were the mean estimated fat mass and fat-free mass.

Findings from long-term non-randomized studies range from comparable to improved weight loss following distal RYGB, but with concerns regarding adverse nutritional effects^6–9^. Major confounders in previous comparative studies were patient selection and lack of standardization of the RYGB intestinal limb lengths.

Distal RYGB has been performed for weight regain after standard RYGB, with improved weight loss^29^. In a systematic review^9^, a long biliopancreatic limb appeared superior to a shorter limb in terms of weight loss after redo surgery. For the present study, a 150-cm common channel in the distal RYGB was used, to reduce the risk of nutritional deficiencies. A shorter common channel may have resulted in greater weight loss, but probably with a risk of severe malabsorption, as previously shown for distal RYGB^29^. The Dutch DUCATI randomized trial^30^^,^^31^ of standard *versus* distal RYGB with a common channel of 100 cm may shed further light on this.

As for RYGB in general, co-morbidities such as type 2 diabetes, hypertension, and dyslipidaemia improved after both procedures. The reductions in fasting serum glucose and HbA1c remained greater after distal RYGB, as also found after 2 years^10^. The change in lipids after distal RYGB are comparable to those reported after other bariatric procedures, with greater reduction of total and LDL-cholesterol, and less of an increase in HDL-cholesterol, compared with standard RYGB^32^. The greater reduction in LDL-cholesterol after distal RYGB is likely to be relevant for cardiovascular risk reduction^33^.

Beside differences relating to nutritional outcomes, adverse events were largely comparable in this study. Internal herniation was more common after standard RYGB, although this result is potentially biased by small numbers. The larger mesenteric defect after distal RYGB may reduce the risk of symptomatic herniation.

Levels of the bone turnover marker CTX-1 and parathyroid hormone increased more after distal than standard RYGB. The prevalence of secondary hyperparathyroidism was also higher after distal compared with standard RYGB, corresponding to findings after 2 years^34^. Higher levels of bone turnover markers are associated with faster bone loss and predict fractures, even after adjustment for aBMD^35–37^. No-between group differences in aBMD were observed at 5 years. However, after distal RYGB three patients had an indication for antiosteoporotic treatment. Distal RYGB may thus have a more substantial negative effect on bone health.

Strengths of this study include the comprehensive evaluations at multiple time points, long-term follow-up, high rate of attendance, and the double-blinded randomized controlled design. Limitations include that the study was not powered to assess differences in the secondary endpoints. The number of patients available for analyses of resting metabolic rate was limited, and these results in particular should be interpreted with caution. Restricting the BMI at study inclusion to 50–60 kg/m^2^ could limit the generalizability of the findings. The entire length of the small intestine was not measured so the total intestinal limb lengths of individual patients is not known.

Elongation of the alimentary limb does not enable increased long-term weight loss after RYGB. The differences observed in other outcomes may not justify routine use of this procedure in patients with a BMI of 50–60 kg/m^2^.

## Funding

The project received research grants from the South-Eastern Norway Regional Health Authority (Health region South-East). O.B.K.S.was granted a research scholarship from the Alexander Malthes Foundation.

## Supplementary Material

zrab105_Supplementary_Data

## References

[1] Adams TD , Davidson LE, Litwin SE, Kolotkin RL, LaMonte MJ, Pendleton RC et al Health benefits of gastric bypass surgery after 6 years. JAMA 2012;308:1122–1131. 22990271 10.1001/2012.jama.11164 PMC3744888

[2] Jakobsen GS , Småstuen MC, Sandbu R, Nordstrand N, Hofsø D, Lindberg M et al Association of bariatric surgery *vs* medical obesity treatment with long-term medical complications and obesity-related comorbidities. JAMA 2018;319:291–301. 29340680 10.1001/jama.2017.21055 PMC5833560

[3] Sturm R , Hattori A. Morbid obesity rates continue to rise rapidly in the United States. Int J Obes (Lond) 2013;37:889–891. 22986681 10.1038/ijo.2012.159 PMC3527647

[4] Risstad H , Søvik TT, Engström M, Aasheim ET, Fagerland MW, Olsén MF et al Five-year outcomes after laparoscopic gastric bypass and laparoscopic duodenal switch in patients with body mass index of 50 to 60: a randomized clinical trial. JAMA Surg 2015;150:352–361. 25650964 10.1001/jamasurg.2014.3579

[5] Søvik TT , Karlsson J, Aasheim ET, Fagerland MW, Björkman S, Engström M et al Gastrointestinal function and eating behavior after gastric bypass and duodenal switch. Surg Obes Relat Dis 2013;9:641–647. 22951078 10.1016/j.soard.2012.06.006

[6] Christou NV , Look D, Maclean LD. Weight gain after short- and long-limb gastric bypass in patients followed for longer than 10 years. Ann Surg 2006;244:734–740. 17060766 10.1097/01.sla.0000217592.04061.d5 PMC1856611

[7] Orci L , Chilcott M, Huber O. Short *versus* long Roux-limb length in Roux-en-Y gastric bypass surgery for the treatment of morbid and super obesity: a systematic review of the literature. Obes Surg 2011;21:797–804. 21479976 10.1007/s11695-011-0409-y

[8] Stefanidis D , Kuwada TS, Gersin KS. The importance of the length of the limbs for gastric bypass patients—an evidence-based review. Obes Surg 2011;21:119–124. 20680504 10.1007/s11695-010-0239-3

[9] Mahawar KK , Kumar P, Parmar C, Graham Y, Carr WRJ, Jennings N, Schroeder N et al Small bowel limb lengths and Roux-en-Y gastric bypass: a systematic review. Obes Surg 2016;26:660–671. 26749410 10.1007/s11695-016-2050-2

[10] Risstad H , Svanevik M, Kristinsson JA, Hjelmesæth J, Aasheim ET, Hofsø D et al Standard *vs* distal Roux-en-Y gastric bypass in patients with body mass index 50 to 60: a double-blind, randomized clinical trial. JAMA Surg 2016;151:1146–1155. 27626242 10.1001/jamasurg.2016.2798

[11] Svanevik M , Risstad H, Hofsø D, Schou CF, Solheim B, Søvik TT et al Perioperative outcomes of proximal and distal gastric bypass in patients with BMI ranged 50–60 kg/m^2^—a double-blind, randomized controlled trial. Obes Surg 2015;25:1788–1795. 25761943 10.1007/s11695-015-1621-y PMC4559572

[12] McConnell DB , O’Rourke RW, Deveney CW. Common channel length predicts outcomes of biliopancreatic diversion alone and with the duodenal switch surgery. Am J Surg 2005;189:536–540. 15862492 10.1016/j.amjsurg.2005.01.023

[13] Blom-Høgestøl IK , Hewitt S, Chahal-Kummen M, Brunborg C, Gulseth HL, Kristinsson JA et al Bone metabolism, bone mineral density and low-energy fractures 10 years after Roux-en-Y gastric bypass. Bone 2019;127:436–445. 31323430 10.1016/j.bone.2019.07.014

[14] GE Healthcare. Reference Data B and Reference Data for adults C. In: Healthcare G (ed.), X-Ray Bone Densitometer with enCore v17 software—User Manual. LU43616NO Revision. Vol. 17. GE Healthcare, 2016, 307–345.

[15] Ware JEJ. SF-36 health survey update. Spine (Phila Pa 1976) 2000;25:3130–3139. 11124729 10.1097/00007632-200012150-00008

[16] Niero M , Martin M, Finger T, Lucas R, Mear I, Wild D et al A new approach to multicultural item generation in the development of two obesity-specific measures: the obesity and weight loss quality of life (OWLQOL) questionnaire and the weight-related symptom measure (WRSM). Clin Ther 2002;24:690–700. 12017412 10.1016/s0149-2918(02)85144-x

[17] Patrick DL , Bushnell DM, Rothman M. Performance of two self-report measures for evaluating obesity and weight loss. Obes Res. 2004;12:48–57. 10.1038/oby.2004.8 14742842

[18] Bjelland I , Dahl AA, Haug TT, Neckelmann D. The validity of the Hospital Anxiety and Depression Scale: an updated literature review. J Psychosom Res 2002;52:69–77. 11832252 10.1016/s0022-3999(01)00296-3

[19] Herrmann C. International experiences with the Hospital Anxiety and Depression Scale—a review of validation data and clinical results. J Psychosom Res 1997;42:17–41. 9055211 10.1016/s0022-3999(96)00216-4

[20] Cappelleri J , Bushmakin AG, Gerber R, Leidy N, Sexton C, Lowe M et al Psychometric analysis of the Three-Factor Eating Questionnaire-R21: results from a large diverse sample of obese and non-obese participants. Int J Obes (Lond) 2009;33:611–620. 19399021 10.1038/ijo.2009.74

[21] Karlsson J , Persson LO, Sjostrom L, Sullivan M. Psychometric properties and factor structure of the Three-Factor Eating Questionnaire (TFEQ) in obese men and women. Results from the Swedish Obese Subjects (SOS) study. Int J Obes Relat Metab Disord 2000;24:1715–1725. 11126230 10.1038/sj.ijo.0801442

[22] Kulich KR , Madisch A, Pacini F, Piqué JM, Regula J, Van Rensburg CJ et al Reliability and validity of the Gastrointestinal Symptom Rating Scale (GSRS) and Quality of Life in Reflux and Dyspepsia (QOLRAD) questionnaire in dyspepsia: a six-country study. Health Qual Life Outcomes 2008;6:12–12. 18237386 10.1186/1477-7525-6-12 PMC2276197

[23] Österberg A , Graf W, Karlbom U, Påhlman L. Evaluation of a questionnaire in the assessment of patients with faecal incontinence and constipation. Scand J Gastroenterol 1996;31:575–580. 8789896 10.3109/00365529609009130

[24] Andersen LF , Tomten H, Haggarty P, Løvø A, Hustvedt BE. Validation of energy intake estimated from a food frequency questionnaire: a doubly labelled water study. Eur J Clin Nutr 2003;57:279–284. 12571660 10.1038/sj.ejcn.1601519

[25] Rosenblad A , Fagerland M, Lydersen S, Laake P. Statistical Analysis of Contingency Tables. Boca Raton, FL: Chapman and Hall/CRC, 2017.

[26] Miras AD , Le Roux CW. Mechanisms underlying weight loss after bariatric surgery. Nat Rev Gastroenterol Hepatol 2013;10:575–584. 23835488 10.1038/nrgastro.2013.119

[27] Laurenius A , Taha O, Maleckas A, Lönroth H, Olbers T. Laparoscopic biliopancreatic diversion/duodenal switch or laparoscopic Roux-en-Y gastric bypass for super-obesity-weight loss *versus* side effects. Surg Obes Relat Dis 2010;6:408–414. 20655023 10.1016/j.soard.2010.03.293

[28] Werling M , Fändriks L, Olbers T, Mala T, Kristinsson J, Stenlöf K et al Biliopancreatic diversion is associated with greater increases in energy expenditure than Roux-en- Y gastric bypass. PLoS One 2018;13:e0194538. 29617391 10.1371/journal.pone.0194538 PMC5884508

[29] Kellum JM , Chikunguwo SM, Maher JW, Wolfe LG, Sugerman HJ. Long-term results of malabsorptive distal Roux-en-Y gastric bypass in superobese patients. Surg Obes Relat Dis 2011;7:189–193. 21145293 10.1016/j.soard.2010.08.018

[30] Gadiot RP , Grotenhuis BA, Biter LU, Dunkelgrun M, Zengerink HJ, Feskens PB et al Study protocol of the DUCATI-study: a randomized controlled trial investigating the optimal common channel length in laparoscopic gastric bypass for morbid obese patients. BMC Obes 2015;2:28. 26217543 10.1186/s40608-015-0059-z PMC4511552

[31] Leeman M , Gadiot R, Wijnand J, Birnie E, Apers J, Biter L et al Effects of standard *versus* very long Roux limb Roux-en-Y gastric bypass on nutrient status: a 1 year follow-up report from the Ducati Study. Br J Nutr 2020;123:1434–1419. 32077402 10.1017/S0007114520000616

[32] Heffron SP , Parikh A, Volodarskiy A, Ren-Fielding C, Schwartzbard A, Nicholson J et al Changes in lipid profile of obese patients following contemporary bariatric surgery: a meta-analysis. Am J Med 2016;129:952–959. 26899751 10.1016/j.amjmed.2016.02.004 PMC4988934

[33] Silverman MG , Ference BA, Im K, Wiviott SD, Giugliano RP, Grundy SM et al Association between lowering LDL-C and cardiovascular risk reduction among different therapeutic interventions: a systematic review and meta-analysis. JAMA 2016;316:1289–1297. 27673306 10.1001/jama.2016.13985

[34] Svanevik M , Risstad H, Hofsø D, Blom-Høgestøl IK, Kristinsson JA, Sandbu R et al Bone turnover markers after standard and distal Roux-en-Y gastric bypass: results from a randomized controlled trial. Obes Surg 2019;29:2886–2895. 31065919 10.1007/s11695-019-03909-1

[35] Marques EA , Gudnason V, Lang T, Sigurdsson G, Sigurdsson S, Aspelund T et al Association of bone turnover markers with volumetric bone loss, periosteal apposition, and fracture risk in older men and women: the AGES-Reykjavik longitudinal study. Osteoporos Int 2016;27:3485–3494. 27341810 10.1007/s00198-016-3675-7 PMC5560053

[36] Johansson H , Kanis JA, Odén A, McCloskey E, Chapurlat RD, Christiansen C et al A meta-analysis of the association of fracture risk and body mass index in women. J Bone Miner Res 2014;29:223–233. 23775829 10.1002/jbmr.2017

[37] Tian A , Ma J, Feng K, Liu Z, Chen L, Jia H et al Reference markers of bone turnover for prediction of fracture: a meta-analysis. J Orthop Surg Res 2019;14:68. 30819222 10.1186/s13018-019-1100-6 PMC6393999

