# Supplementary material for: Standard versus distal Roux-en-Y gastric bypass in patients with BMI 50–60 kg/m2: 5-year outcomes of a double-blind, randomized clinical trial
Source: BJS Open. 2021 Nov 17;5(6):zrab105. doi: 10.1093/bjsopen/zrab105 (PMC8599874; doi:10.1093/bjsopen/zrab105)
Supplement: zrab105_Supplementary_Data [file zrab105_supplementary_data.zip › Supplementary_material.docx]

**Supplementary material**

***Table S1.*** *Trial definitions of comorbidities and their resolutions.*

***Table S2.*** *Areal bone mineral density (aBMD) 5 years after standard and distal Roux-en-Y gastric bypass (RYGB).*

***Table S3.*** *Bone turnover markers, PTH (parathyroid hormone) and calcium levels for 113 patients before and 5 years after standard (n=57) and distal (n=56) Roux-en-Y gastric bypass (RYGB).* *Linear mixed models were used for analyses.*

***Table S4.*** *Short Form 36 Health Survey (SF-36) scores before and after standard (n=57) and distal (n=56) Roux-en-Y gastric bypass.* *Linear mixed models were used for analyses.*

***Figure S1.*** *SF-36 radar plots with estimated mean summary scores before and 5 years after standard and distal Roux- en-Y gastric bypass (RYGB) (linear mixed models). At baseline 57 patients received standard and 56 patients distal RYGB. At 5 years 48 and 44 patients attended follow-up, respectively.*

***Table S5.*** *Three-Factor Eating Questionnaire-R 21 (TFEQ) and HADs (Hospital Anxiety and Depression scale) scores 5 years after standard and distal Roux-en-Y gastric bypass. At baseline 57 patients received standard and 56 patients distal RYGB.* *At 5 years 48 and 44 patients attended follow-up, respectively. Linear mixed models were used for analyses.*

***Table S6.*** *Gastro-intestinal symptoms score (GSRS) 5 years after standard and distal Roux-en Y gastric bypass (RYGB).*

***Table S7.*** *Daily intake of energy yielding nutrients 5 years after standard and distal Roux-en-Y gastric bypass (RYGB)*

***Table S8.*** *Resting metabolic rate 5 years after standard and distal Roux-en-Y gastric bypass (RYGB).*

This supplementary material provides additional information about the submitted work.

***Table S1.*** *Trial definitions of comorbidities and their resolutions.*

| ***Type 2 diabetes:*** | - Fasting glucose ≥126 mg/dL( ≥ 7.0 mmol/L) - HbA1c ≥ 48 mmol/mol (≥ 6.5 %) - And/or use of antidiabetic medications - Absence of type 1 diabetes |
| --- | --- |
| ***Hypertension:*** | - Systolic BP ≥ 140 mmHg - Diastolic BP ≥ 90 mmHg - And/or use of antihypertensive drugs |
| ***Secondary hyperparathyroidism:*** | - PTH ≥ 66 pg/ml (≥ 7.0 pmol/L) *without* hypercalcemia - Hypercalcemia: free/ionized calcium ≥ 5.16 mg/dL (≥ 1.29 mmol/L) |
| ***Iron deficiency:*** | - Ferritin ≤ 20 ng/ml - Iron ≤ 50 g/µdL (≤ 9 µmol/L) - And/or those with anemia |
| ***Anemia*** | - Hemoglobin ≤ 13.5 g/dL in men, ≤ 12.0 g/dL in women - And/or need for iron infusion and/or blood transfusion |
| ***Dyslipidemia*** | - LDL cholesterol ≥ 116 mg/dL (≥ 3.0 mmol/L) - HDL < 40 mg/dL (< 1.03 mmol/L) in men and < 50 mg/dL (< 1.29 mmol/L) in women - Triglycerdies ≥ 150 mg/dL ( ≥ 1.7 mmol/L) - Total cholesterol /HDL cholesterol ratio > 5.0 - And/or use of lipid lowering medications |
| ***Vitamin deficiencies*** | - Vitamin A < 10 µg/dL (< 0.35 µmol/L) (ref. 20-80 µg/dL) - Vitamin B1 (Thiamine) < 70 nmol/L (ref 95-200 nmol/L) - Vitamin B9 (Folate) < 3 ng/mL (<7 nmol/L) (ref. 340-1020 ng/mL) - Vitamin B12 < 200 pg/mL (< 150 pmol/L) ( ref 200-1000 pg/mL ) - Vitamin D (25 OH) < 20 ng/mL (< 50nmol/L ) (ref >30 ng/mL) - And/or increased substitution therapy |
| ***Metabolic syndrome definition:***  *(International Diabetes Federation)* | **Central obesity**  (waist circumference – European: ≥ 94 cm males, ≥ 80 cm females) or BMI ≥ 30 (then central obesity is assumed) + 2 or more of any of the following:   1. Triglycerides ≥ 150 mg/dL (≥ 1.7 mmol/l) 2. HDL cholesterol < 40 mg/dL (< 1.03 mmol/L) in males, < 50 mg/dL   (< 1.29 mmol/L) in females   1. Raised blood pressure (≥ 130 mmHg systolic BP and/or ≥ 85 mmHg diastolic BP) or on antihypertensive medication 2. Fasting glucose ≥100 mg/dL (≥ 5.6 mmol/L) or diagnosis of type 2 diabetes |
| ***Osteoporosis*** | - DEXA: t-score ≤ -2.5 of lumbar spine, femoral neck and/or hip |
| ***Resolution of Type 2 diabetes:*** | - HbA1c ≤ 42 mmol/mol (≤ 6.0 %) - And no use of antidiabetic medication |
| ***Resolution of hypertension*** | - - Systolic BP < 140 mmHg   - And Diastolic BP < 90 mmHg   - And no use of antihypertensive medication |

***Table S2.*** *Areal bone mineral density (aBMD) 5 years after standard and distal Roux-en-Y gastric bypass (RYGB).*

|  | **Standard RYGB** (n=43)  Mean (95% CI) | **Distal RYGB**  (n=39)  Mean (95% CI) | **Between-group difference**  mean (95% CI); p-value | |
| --- | --- | --- | --- | --- |
| aBMD (L_1_-L_4_) | 1.16 (1.12 to 1.21) | 1.12 (1.06 to 1.18) | -0.04 (-0.11 to 0.29) | P =0.25 |
| t-score (L_1_-L_4_) | 0.35 (0.00 to 0.70) | -0.06 (-0.50 to 0.39) | -0.04 (-0.96 to 0.14) | P =0.15 |
| z-score(L_1_-L_4_) | 0.24 (-0.17 to 0.66) | -0.28 (-0.75 to 0.19) | -0.52 (-1.14 to 0.09) | P =0.10 |
| aBMD (left proximal femur) | 0.36 (0.91 to 1.00) | 0.94 (0.89 to 0.99) | -0.02 (-0.08 to 0.05) | P =0.64 |
| t-score (left proximal femur) | -0.01 (-0.32 to 0.31) | -0.18 (-0.55 to 0.19) | -0.17 (-0.65 to 0.30) | P =0.47 |
| z-score (left proximal femur) | 0.25 (-0.08 to 0.57) | 0.04 (-0.35 to 0.42) | -0.21 (-0.70 to 0.28) | P =0.40 |
| aBMD (left hip) | 1.03 (0.99 to 1.10) | 1.02 (0.97 to 1.07) | -0.014 (-0.08 to 0.05) | P =0.68 |
| t-score (left hip) | 0.45 (0.11 to 0.79) | 0.25 (-0.10 to 0.61) | -0.20 (-0.68 to 0.29) | P =0.42 |
| z-score (left hip) | 0.36 (0.02 to 0.70) | 0.12 (-0.24 to 0.47) | -0.25 (-0.72 to 0.23) | P =0.31 |

P-values are based on independent t-test between standard and distal RYGB. The results are given as mean (95% CI) for continuous variables with normal distribution.

***Table S3.*** *Bone turnover markers, PTH (parathyroid hormone) and calcium levels for 113 patients before and 5 years after standard (n=57) and distal (n=56) Roux-en-Y gastric bypass (RYGB).* *Linear mixed models were used for analyses.*

|  | **Baseline**  mean (95% CI)  Standard n=57  Distal n=56 | **2 years**  mean (95% CI)  Standard n=55  Distal n=55 | **5 years**  mean (95% CI)  Standard n=48  Distal n=44 | **Change from baseline to 5 years**  mean (95% CI) | **Between-group difference in changes from baseline to 5 years**  mean (95% CI); p-value |
| --- | --- | --- | --- | --- | --- |
| **CTX-1**, µg/L | | | | | |
| Standard | 0.37 (0.30 to 0.45) | 0.79 (0.72 to 0.86) | 0.54 (0.47 to 0.62) | 0.17 (0.09 to 0.25) | 0.19  (0.07 to 0.30);  **P =0.002** |
| Distal | 0.30 (0.22 to 0.38) | 0.86 (0.78 to 0.93) | 0.66 (0.58 to 0.74) | 0.36 (0.27 to 0.44) |  |
| **P1NP**, µg/L | | | | | |
| Standard | 44.0 (37.0 to 51.0) | 76.0 (69.2 to 82.8) | 68.1 (60.7 to 75.4) | 24.1 (16.4 to 31.7) | 5.8  (-5.2 to 16.8);  P =0.30 |
| Distal | 40.3 (33.2 to 47.4) | 79.2 (72.3 to 86.1) | 70.2 (62.7 to 77.7) | 29.9 (22.0 to 37.8) |  |
| **BALP**, µg U/L | | | | | |
| Standard | 32.3 (29.6 to 35.1) | 24.7 (21.8 to 27.5) | 16.6 (13.5 to 19.6) | -15.7 (-19.0 to -12.5) | 0.1  (-4.6 to 4.8);  P =0.98 |
| Distal | 32.9 (30.1 to 35.7) | 26.9 (24.0 to 29.7) | 17.2 (14.0 to 20.4) | -15.7 (-19.1 to -12.3) |  |
| **PTH**, pg/mL | | | | | |
| Standard | 59.4 (48.1 to 69.8) | 59.4 (48.1 to 70.7) | 67.0 (54.7 to 78.3) | 7.5 (-3.8-to 18.9) | 28.3  (12.3 to 44.3);  **P =0.001** |
| Distal | 53.8 (43.4 to 65.1) | 80.2 (68.8 to 91.5) | 90.5 (78.3 to 101.8) | 35.8 (24.5 to 47.2) |  |
| **Calcium**, free, mg/dL | | | | | |
| Standard | 4.80 (4.68 to 4.92) | 4.80 (4.68 to 4.92) | 4.76 (4.64 to 4.88) | -0.04 (-0.20 to 0.12) | 0.08  (-0.16 to 0.36);  P =0.44 |
| Distal | 4.68 (4.56 to 4.80) | 4.76 (4.64 to 4.88) | 4.72 (4.60 to 4.88) | 0.04 (-0.12 to 0.24) |  |

Abbreviations, Carboxyl terminal telopeptide of type 1 collagen (CTX-1), Procollagen type 1 N-terminal propeptide (P1NP), Bone specific alkaline phosphatase (BALP). Parathyroid hormone (PTH), Reference ranges: *CTX-1 µg/L*: females 25-49 years: ≤ 0.57, ≥50 years: ≤1.01, males 30-50 years: ≤ 0.58 , 51-70 years: ≤ 0.7; *P1NP µg/L*: females >25 years: 11-94, males >25 years: 20-91; *BALP* *µg/L:* 5.5-24.6; PTH pg/mL: 14.1-66.0; Free calcium mg/dL: 4.60-5.32 (1.15-1.33 *mmol/L)*

***Table S4.*** *Short Form 36 Health Survey (SF-36) scores before and after standard (n=57) and distal (n=56) Roux-en-Y gastric bypass.* *Linear mixed models were used for analyses.*

|  | **Baseline**  mean (95% CI)  Standard n=57  Distal n=56 | **2 years**  mean (95% CI)  Standard n=55  Distaln n=55 | **5 years**  mean (95% CI)  Standard n=48  Distal n=44 | **Change from baseline to 5 years**  mean (95% CI) | **Between-group difference in changes from baseline to 5 years**  mean (95% CI); P-value |
| --- | --- | --- | --- | --- | --- |
| **Short Form Health Survey 36** | | | | | |
| **Physical Component Summary** | | | | | |
| Standard | 38.1 (35.6 to 40.7) | 49.1 (46.5 to 51.7) | 47.8 (45.1 to 50.5) | 9.6 (7.1 to 12.1) | -0.5 (- 4.1 to 3.1); P =0.77 |
| Distal | 35.7 (33.1 to 38.2) | 50.1 (47.6 to 52.7) | 44.8 (42.0 to 47.6) | 9.1 (6.5 to 11.7) |  |
| **Mental Component Summary** | | | | | |
| Standard | 49.1 (46.2 to 52.0) | 50.8 (47.8 to 53.8) | 50.4 (47.3 to 53.5) | 1.3 (-1.9 to 4.5) | -0.6 (-5.3 to 4.0); P =0.80 |
| Distal | 48.1 (45.1 to 51.0) | 49.8 (46.9 to 52.8) | 48.8 (45.5 to 52.1) | 0.7 (-2.7 to 4.1) |  |
| **Physical Function** | | | | | |
| Standard | 39.9 (37.9 to 41.9) | 52.2 (50.2 to 54.2) | 50.5 (48.4 to 52.6) | 10.5 (8.5 to 12.5) | 0.9 (-1.9 to 3.8); P =0.52 |
| Distal | 38.2 (36.2 to 40.2) | 52.0 (50.0 to 54.0) | 49.6 (27.4 to 51.8) | 11.5 (9.4 to 13.5) |  |
| **Role Physical** | | | | | |
| Standard | 41.9 (39.1 to 44.7) | 49.4 (46.6 to 52.2) | 49.4 (46.4 to 52.4) | 7.5 (4.4 to 10.5) | -3.2 (7.6 to 1.2); P =0.16 |
| Distal | 39.7 (36.9 to 42.5) | 49.2 (46.4 to 52.0) | 44.0 (40.8 to 47.1) | 4.3 (1.1 to 7.5) |  |
| **Bodily Pain** | | | | | |
| Standard | 41.3 (38.1 to 44.6) | 45.5 (42.2 to 48.8) | 44.6 (41.1 to 48.1) | 3.3 (-0.1 to 6.7) | 1.0 (-3.9 to 5.9); P =0.69 |
| Distal | 37.4 (34.1 to 40.7) | 46.1 (42.8 to 49.4) | 41.7 (38.1 to 45.4) | 4.3 (0.7 to 7.8) |  |
| **Mental Health** | | | | | |
| Standard | 48.7 (45.9 to 51.4) | 51.7 (49.0 to 54.5) | 50.8 (47.9 to 53.7) | 2.2 (-0.8 to 5.0) | -1.0 (-5.2 to 3.3); P =0.66 |
| Distal | 48.9 (46.1 to 51.7) | 50.3 (47.5 to 53.1) | 50.1 (47.0 to 53.1) | 1.2 (-1.9 to 4.2) |  |
| **Role Emotional** | | | | | |
| Standard | 47.6 (44.8 to 50.5) | 50.6 (47.7 to 53.5) | 51.7 (48.6 to 54.8) | 4.0 (0.6 to 7.5) | -0.5 (-5.5 to 4.5);  P = 0.84 |
| Distal | 43.8 (40.9 to 46.7) | 47.6 (44.7 to 50.5) | 47.3 (44.1 to 50.6) | 3.5 (-0.1 to 7.1) |  |
| **Social Function** | | | | | |
| Standard | 42.3 (39.5 to 45.0) | 48.6 (45.8 to 51.4) | 48.2 (45.3 to 51.1) | 6.0 (2.8 to 9.1) | -1.2 (-5.7 to 3.3); P =0.59 |
| Distal | 42.7 (39.9 to 45.5) | 50.4 (47.6 to 53.1) | 47.4 (44.3 to 50.5) | 4.7 (1.5 to 8.0) |  |
| **Vitality** | | | | | |
| Standard | 43.5 (40.6 to 46.4) | 50.4 (47.5 to 53.4) | 47.1 (33.0 to 50.1) | 3.6 (0.7 to 6.5) | -0.2 (-4.4 to 4.1); P =0.94 |
| Distal | 42.2 (39.3 to 45.1) | 51.5 (48.4 to 54.4) | 45.6 (42.5 to 48.8) | 3.4 (0.4 to 6.5) |  |
| **General Health** | | | | | |
| Standard | 39.5 (36.6 to 42.3) | 51.4 (48.6 to 54.3) | 50.2 (47.2 to 53.2) | 10.7 (8.2 to 13.3) | -2.3 (-6.0 to 1.4); P =0.22 |
| Distal | 38.8 (35.9 to 41.7) | 51.3 (48.4 to 54.1) | 47.2 (44.1 to 50.3) | 8.4 (5.7 to 11.1) |  |
| **Obesity and Weight-Loss Quality of Life Instrument** | | | | | |
| Standard | 37.7 (32.4 to 42.9) | 77.8 (72.5 to 83.1) | 76.3 (70.7 to 81.8) | 38.6 (33.4 to 43.8) | -2.3 (-9.8 to 5.2); P = 0.55 |
| Distal | 35.3 (30.0 to 40.6) | 74.3 (69.0 to 79.6) | 71.6 (65.8 to 77.3) | 36.3 (30.9 to 41.7) |  |
| **Weight Related Symptoms Measure** | | | | | |
| **Symptom Count** | | | | | |
| Standard | 9.4 (8.2 to 10.6) | 6.9 (5.7 to 8.1) | 6.7 (5.4 to 7.9) | -2.8 (-3.9 to -1.5) | 0.0 (-1.8 to 1.8); P = 0.97 |
| Distal | 10.2 (9.0 to 11.4) | 6.4 (5.1 to 7.6) | 7.4 (6.1 to 8.7) | -2.8 (-4.1 to -1.5) |  |
| **Distress Score** | | | | | |
| Standard | 32.6 (28.1 to 37.1) | 19.8 (15.5 to 24.1) | 18.1 (13.4 to 22.7) | -14.5 (-18.9 to -10.0) | 0.5 (-5.9 to 6.8); P = 0.89 |
| Distal | 36.3 (31.8 to 40.9) | 18.5 (14.1 to 22.9) | 22.3 (17.6 to 27.0) | -14.0 (-18.5 to -9.5) |  |

***Figure S1.*** *SF-36 radar plots with estimated mean summary scores before and 5 years after standard and distal Roux- en-Y gastric bypass (RYGB) (linear mixed models). At baseline 57 patients received standard and 56 patients distal RYGB. At 5 years 48 and 44 patients attended follow-up, respectively.*

SF-36: Short Form 36 Health Survey. Radar plot displaying precentages for each SF-36 parameter for baseline, 2- and 5-years after standard and distal RYGB. * Significant change from baseline to 5-year follow-up. No between-group differences were observed.

***Table S5.*** *Three-Factor Eating Questionnaire-R 21 (TFEQ) and HADs (Hospital Anxiety and Depression scale) scores 5 years after standard and distal Roux-en-Y gastric bypass. At baseline 57 patients received standard and 56 patients distal RYGB. At 5 years 48 and 44 patients attended follow-up, respectively. Linear mixed models were used for analyses.*

|  | **5 years**  **Mean (SD)** | **Difference between groups**  **Mean (95% CI)** | **P-value** |
| --- | --- | --- | --- |
| **TFEQ** |  |  |  |
| **Uncontrolled Eating** |  |  |  |
| Standard | 24.6 (19.7 to 29.5) | -8.2 (-17.2 to 0.7) | P=0.07^#^ |
| Distal | 32.8 (24.9 to 40.7) |  |  |
|  |  |  |  |
| **Cognitive Restraint** |  |  |  |
| Standard | 52.0 (45.9 to 58.1) | 4.9 (-3.5 to 13.3) | P=0.25^#^ |
| Distal | 47.1 (41.2 to 53.1) |  |  |
|  |  |  |  |
| **Emotional Eating** |  |  |  |
| Standard | 31.2 (23.9 to 38.5) | -5.6 (-16.8 to 5.6) | P=0.32^#^ |
| Distal | 36.8 (28.0 to 45.7) |  |  |
| **HAD**  **Anxiety** |  |  |  |
| Standard | 4.7 (3.4 to 6.0) | -1.1 (-3.1 to 0.8) | P=0.20^#^ |
| Distal | 5.8 (4.3 to 7.4) |  |  |
|  |  |  |  |
| **Depression** |  |  |  |
| Standard | 2.5 (1.4 to 3.7) | -1.1 (-2.7 to 0.5) | P=0.17^#^ |
| Distal | 3.6 (2.5 to 4.8) |  |  |
|  |  |  |  |
| **Clinical anxiety** | **No. (%)** |  |  |
| Standard | 11(24.4) | n/a | P=0.34^*^ |
| Distal | 14 (35.9) |  |  |
|  |  |  |  |
| **Clinical depression** |  |  |  |
| Standard | 4 (8.9) | n/a | P=0.21^*^ |
| Distal | 8 (20.5) |  |  |

Estimated mean scores with 95% confidence intervals, ^#^independent samples t-test. ^*^Fisher’s exact test

| **GSRS symptom score** | **Standard RYGB**  **Mean (95% CI)**  **(n=42)** | **Distal RYGB**  **Mean (95% CI)**  **(n=47)** | **Difference between groups**  **Mean (95% CI)** | **P-value** |
| --- | --- | --- | --- | --- |
| **Diarrhea** | 2.1 (1.7 to 2.5) | 2.8 (2.3 to 3.3) | -0.72 (-1.32 to -0.13) | **0.02** |
| **Indigestion** | 2.5 (2.1 to 2.8) | 2.8 (2.5 to 3.2) | -0.38 (-0.87 to 0.17) | 0.13 |
| **Constipation** | 2.0 (1.7 to 2.3) | 1.8 (1.5 to 2.0) | 0.23 (-0.19 to 0.65) | 0.27 |
| **Abdominal pain** | 2.1 (1.8 to 2.4) | 2.3 (2.0 to 2.6) | -0.16 (-0.58 to 0.25) | 0.43 |
| **Reflux** | 1.2 (1.1 to 1.4) | 1.3 (1.0 to 1.5) | -0.02 (-0.32 to 0.30) | 0.92 |

***Table S6.*** *Gastro-intestinal symptoms score (GSRS) 5 years after standard and distal Roux-en Y gastric bypass (RYGB).*

***Table S7.*** *Daily intake of energy yielding nutrients 5 years after standard and distal Roux-en-Y gastric bypass (RYGB)*

| **Nutrient** | **RDI^1^** | **Standard RYGB**  **Mean (95% CI)**  **(n=44)** | **Distal RYGB**  **Mean (95% CI)**  **(n=39)** | **Mean Difference (95% CI)** | **P-value** |
| --- | --- | --- | --- | --- | --- |
| **Energy (MJ)** | 9-11^2^ | 8.2 (7.0 to 9.0) | 9.8 (8.4 to 11.2) | -1.6 (-3.4 to 0.1) | 0.07 |
| **Energy (kcal)** |  | 1952 (1684 to 2221) | 2341 (2003 to 2681) | -390 (-811 to 32) | 0.07 |
| **Protein (g)** |  | 79 (70 to 87) | 96 (84 to 108) | -17 (-32 to -3) | 0.02 |
| **Protein (E%)** | 10-20 | 17 (16 to 18) | 17 (16 to 18) | -0.1 (-1.6 to 1.4) | 0.88 |
| **Total fat (g)** |  | 78 (67 to 89) | 91 (80 to 103) | -13.4 (-28.8 to 2) | 0.09 |
| **Total fat (E%)** | 25-40 | 36 (34 to 39) | 36 (33 to 39) | 0.1 (-3.8 to 3.9) | 0.97 |
| **SFA (E%)** | <10 | 13 (12 to 14) | 14 (12 to 15) | -0.5 (-2.3 to 1.4) | 0.63 |
| **MUFA E%)** | 10-20 | 14 (13 to 15) | 14 (13 to 15) | -0.2 (-1.7 to 1.4) | 0.83 |
| **PUFA (E%)** | 5-10 | 7 (6 to 8) | 6 (5 to 7) | 0.7 (-0.4 to 1.7) | 0.21 |
| **CHO (g)** |  | 213 (174 to 251) | 245 (207 to 283) | -33 (-86 to 21) | 0.23 |
| **CHO (E%)** | 45-60 | 42 (40 to 45) | 42 (39 to 45) | 0.6 (-3.1 to 4.3) | 0.75 |
| **Sugar (E%)** | <10 | 6 (4 to 8) | 6 (5 to 8) | -0.8 (-3.3 to 1.8) | 0.55 |
| **Fiber (g)** | ≥25^3^ | 28 (23 to 33) | 28 (25 to 31) | -0.4 (-6.5 to 5.8) | 0.27 |
| **Fiber (E%)** |  | 2.7 (2.5 to 3) | 2.5 (2.2 to 2.7) | 0.3 (-0.1 to 0.6) | 0.11 |
| **Alcohol (g)** | <10^4^ | 4 (2 to 6) | 14 (0.0 to 28.9) | -9.8 (-23.9 to 4.2) | 0.20 |
| **Alcohol E%** | <5 | 1.5 (0.8 to 2.3) | 2.4 (0.6 to 4.2) | -0.9 (-2.7 to 1.0) | 0.35 |

*^1^*Recommended Daily Intake (RDI).

^2^The individual mean recommended daily energy intake is dependent on gender, age and physical activity level. For normal weight individuals (BMI 18.5-25) aged 31-60 years with a sedentary lifestyle, energy intake reference is 9 MJ/day for women and 11 MJ/day for men.

^3^RDI ≥25/day for women and ≥35 g/day for men.

^4^<10 g/day for women, <20 g/day for men. Pregnant and lactating women are recommended no alcohol intake.

SFA, saturated fatty acids; MUFA, monounsaturated fatty acids; PUFA, polyunsaturated fatty acids; CHO, carbohydrates.

***Table S8.*** *Resting metabolic rate 5 years after standard and distal Roux-en-Y gastric bypass (RYGB).*

|  | **Standard RYGB**  **(n =20)** | **Distal RYGB**  **(n =17)** | **Differences**  **between groups** | **P value** |
| --- | --- | --- | --- | --- |
| **Female** | 15 (75%) | 12 (71%) |  | 0.76 |
| **Body weight (kg)** | 115.4 (109.1 to 121.7) | 109.5 (100.0 to 119.0) | 5.9 (-4.7 to 16.6) | 0.27 |
| **RMR**  **(Kcal·day^-1^)** | 2492.9 (2206.8 to 2779.0) | 2267.5 (2139.8 to 2395.2) | 225.4 (-81.6 to 532.5) | 0.14 |

Data presented as numbers (%) or mean (95% CI), independent samples t-test. Roux- en-Y gastric bypass, RMR; resting metabolic rate. Metabolic rate was measured at Vestfold Hospital Trust.
